# Use of Personal Hearing Protection Devices at Loud Athletic or Entertainment Events Among Adults — United States, 2018

**DOI:** 10.15585/mmwr.mm6741a4

**Published:** 2018-10-19

**Authors:** John Eichwald, Franco Scinicariello, Jana L. Telfer, Yulia I. Carroll

**Affiliations:** ^1^Office of Science, National Center for Environment Health, CDC; ^2^Division of Toxicology and Human Health Services, Agency for Toxic Substances and Disease Registry, Atlanta, Georgia; ^3^Office of the Director, National Center for Environment Health, CDC.

Tens of millions of U.S. residents have a range of adverse health outcomes caused by noise exposure (*1*). During 2011–2012, 21 million U.S. adults who reported no exposure to loud or very loud noise at work exhibited hearing damage suggestive of noise-induced hearing loss (*2*). In addition to the known risk for hearing damage, nonauditory adverse health outcomes and health risks from excessive environmental sound exposure can include effects on the cardiovascular system, metabolism, blood pressure, body weight, cognition, sleep, mental health, quality of life, and overall well-being (*1*,*3*,*4*). CDC analyzed a representative sample of the U.S. adult population (aged ≥18 years) from a 2018 national marketing survey (50 states and the District of Columbia) that included questions about use of hearing protection devices (HPDs) (e.g., ear plugs or ear muffs) during recreational exposure to loud athletic and entertainment events; approximately 8% of respondents reported consistent use of an HPD at these types of events. Among those adults more likely to wear an HPD, 63.8% had at least some college education, and 49.1% had higher income levels. Women and older adults were significantly less likely to use HPDs. These findings suggest a need to strengthen a public health focus on the adverse health effects of excessive noise exposure at home and in recreational settings as well as a need for continued efforts to raise public awareness about the protective value of HPDs.

Sound intensity at recreational events can reach hazardous levels and might remain high for the duration of the event, thereby increasing the risk for hearing damage. To protect the public health and welfare, in 1974 the Environmental Protection Agency determined that a 24-hour exposure limit level of 70 decibels (dB) would produce minimal hearing loss in 96% of the population.* In 1999, the World Health Organization Guidelines for Community Noise concluded that a 24-hour equivalent sound level of ≤70 dB would avoid hearing impairment in 95% of persons, even over a lifetime of exposure.^†^

In an assessment of noise exposure at college basketball games, attendees wearing dosimeters at a midsized arena were exposed to average sound levels over 98 dB, with peak levels ranging from 127.5 to 138.3 dB (*5*). Other investigators reported sound level measurements at arenas hosting hockey games ranging from 81 to 96 dB, with peak sound levels from 105 to 124 dB (*6*). In another investigation, recorded instantaneous peak sound levels of up to 140 dB during college football games were reported (*7*). As recommended by the National Hearing Conservation Association, persons exposed to high levels of sounds can limit their risk by using a personal HPD, increasing distance from the source, and by taking quiet breaks to reduce their overall sound exposure (*8*).

CDC analyzed data from the 2018 SpringStyles, a cross-sectional, national online marketing survey conducted by Porter Novelli via the KnowledgePanel of the market research firm Growth for Knowledge.^§^ Panel members were randomly recruited by mail using probability-based sampling by address to reach respondents regardless of whether they had landline telephones or Internet access. If needed, households were provided with a laptop or tablet computer and Internet access. During March 21–April 11, 2018, a random sample of 10,904 panelists received an initial SpringStyles survey covering a wide range of personal health-related conditions, knowledge, and attitudes. Panelists who did not answer at least half of the questions or who completed the survey in ≤5 minutes were removed, resulting in a response rate of 58.9%. Panelists who completed the survey received a cash-equivalent reward worth approximately $5. To match U.S. population proportions, participant responses were weighted to March 2017 U.S. Census estimates on eight selected demographic variables: age, census region, education, sex, household income, household size, metro status, and race/ethnicity.

The 2018 SpringStyles survey included the following question related to the use of an HPD during recreational exposure to loud sounds: “In the past 12 months, how often did you wear hearing protection devices (ear plugs, ear muffs) when attending a loud athletic or entertainment event?*”* Participants were asked to indicate their responses on a 5-point Likert scale (never or seldom, some of the time, about half the time, most of the time, or always).

Independent variables included sex, age, race/ethnicity, education, household income, metropolitan statistical area of residence status, presence of hearing impairment in a household member, and frequent sporting event attendance. A total of 6,357 adults answered the question concerning HPD use during a loud athletic or entertainment event. Researchers combined participant answers into three categories: never or seldom, some or about half the time, and most of the time or always; they then applied adjusted multinomial logistic regression to examine how the likelihood of wearing an HPD varied by sociodemographic factors.

Overall, 81.8% of U.S. adults aged ≥18 years reported never or seldom wearing an HPD when attending a loud athletic or entertainment event (Table 1). The majority of adults who never or seldom wore HPDs at these types of events were women (54.4%), white (65.1%), or lived in a metropolitan area (86.5%). Adults who were more likely to wear an HPD (most of the time or always) at loud athletic or entertainment events had at least some college education (63.8%) or had household incomes of ≥$75,000 (49.1%).

**TABLE 1:** Selected characteristics regarding the use of personal hearing protection devices (HPDs) when attending a loud athletic or entertainment event in the past 12 months among adults aged ≥18 years — Porter Novelli SpringStyles panelists, United States, 2018

Characteristic	Unweighted no.	Weighted no.	All respondents	Never or seldom	Some or about half the time	Most of the time or always
			Weighted % (95% CI)	Weighted % (95% CI)	Weighted % (95% CI)	Weighted % (95% CI)
HPD use*						
Never or seldom	5,247	5,197	81.84 (80.64–83.03)	**—**	**—**	**—**
Some or about half the time	591	6,410	10.08 (9.12–11.03)	**—**	**—**	**—**
Most of the time or always	519	514	8.08 (7.25–8.91)	**—**	**—**	**—**
All respondents	6,357	6,351	**—**	81.84 (80.64–83.03)	10.08 (9.12–11.03)	8.08 (7.25–8.91)
**Sex**						
Men	2,874	3,066	48.28 (46.78–49.79)	45.63 (43.99–47.27)	60.22 (55.37–65.08)	60.25 (55.03–65.47)
Women	3,483	3,284	51.72 (50.21–53.22)	54.37 (52.73–56.01)	39.78 (34.92–44.63)	39.75 (34.53–44.97)
**Age group (yrs)**						
18–24	236	697	10.97 (9.65–12.30)	10.14 (8.73–11.56)	16.28 (11.45–21.10)	12.77 (7.76–17.78)
25–34	800	1,149	18.09 (16.84–19.33)	17.47 (16.12–18.82)	21.57 (17.20–25.93)	20.06 (15.45–24.67)
35–44	1,247	1,044	16.44 (15.38-–17.49)	16.00 (14.87–17.14)	20.29 (16.43–24.15)	16.04 (12.20–19.89)
45–54	1,515	1,100	17.32 (16.30–18.34)	17.76 (16.62–18.91)	13.72 (10.86–16.58)	17.35 (13.87–20.83)
55–64	1,318	1,078	16.97 (16.00–17.95)	17.32 (16.23–18.40)	14.70 (11.78–17.61)	16.34 (13.07–19.62)
65–74	863	901	14.19 (13.23–15.14)	14.68 (13.61–15.74)	10.84 (8.04–13.64)	13.42 (10.19–16.66)
≥75	378	382	6.02 (5.39–6.64)	6.63 (5.90–7.36)	2.61 (1.31–3.91)	4.01 (2.25–5.78)
**Race/Ethnicity^†^**						
White	4,719	4,100	64.55 (63.01–66.10)	65.12 (63.43–66.80)	55.99 (50.81–61.17)	69.51 (64.05–74.98)
Black	537	741	11.66 (10.60–12.73)	11.52 (10.38–12.66)	14.41 (10.37 −18.44)	9.68 (5.82–13.55)
Hispanic	576	851	13.39 (12.20–14.59)	12.72 (11.44–14.00)	18.76 (14.18–23.35)	13.52 (9.24–17.79)
Asian	214	340	5.35 (4.56 −6.14)	5.73 (4.82–6.64)	4.41 (2.30–6.52)	2.66 (0.71–4.61)
Other, multiracial	311	320	5.04 (4.31–5.77)	4.91 (4.11–5.71)	6.43 (3.79–9.07)	4.63 (2.30–6.96)
**Education**						
High school or less	1,755	2,496	39.30 (37.76–40.85)	39.67 (37.99–41.36)	38.83 (33.62–44.04)	36.16 (30.51–41.81)
Some college or associate degree	1,967	1,827	28.78 (27.46–30.10)	28.99 (27.54–30.44)	29.59 (25.08–34.11)	25.62 (21.24–30.00)
Bachelor's degree or higher	2,635	2,027	31.92 (30.63–33.21)	31.34 (29.93–32.75)	31.57 (27.38–35.77)	38.22 (33.31–43.14)
**Income**						
<$40,000	1,522	1,712	26.96 (25.58–28.35)	26.48 (24.99–27.96)	31.57 (26.61–36.53)	26.15 (20.90–31.39)
$40,000–$74,999	1,627	1,626	25.61 (24.30–26.92)	26.33 (24.88–27.79)	20.40 (16.47–24.33)	24.79 (20.15–29.43)
$75,000–$124,999	1,828	1,715	27.00 (25.69–28.31)	26.82 (25.37–28.26)	25.35 (21.12–29.57)	30.94 (26.20–35.67)
≥$125,000	1,380	1,297	20.42 (19.26–21.59)	20.37 (19.09–21.66)	22.68 (18.64–26.73)	18.13 (14.39–21.86)
**U.S. Census region of residence^§^**						
Northeast	1,137	1,132	17.82 (16.68–18.95)	18.53 (17.26–19.79)	15.41 (11.86–18.96)	13.60 (9.96–17.24)
Midwest	1,573	1,335	21.02 (19.86–22.18)	21.41 (20.12–22.70)	20.25 (16.46–24.04)	18.03 (14.33–21.74)
South	2,224	2,380	37.47 (36.00–38.94)	36.99 (35.39–38.59)	38.53 (33.58–43.48)	41.00 (35.62–46.38)
West	1,423	1,505	23.69 (22.39–25.00)	23.07 (21.65–24.49)	25.81 (21.27–30.34)	27.36 (22.57–32.16)
**Metropolitan statistical area status**						
Nonmetropolitan	898	885	13.93 (12.92–14.95)	13.54 (12.46–14.63)	14.31 (10.88–17.75)	17.39 (13.18–21.60)
Metropolitan	5,459	5,466	86.07 (85.05–87.08)	86.46 (85.37–87.54)	85.69 (82.25–89.12)	82.61 (78.40–86.82)
**Household hearing impairment^¶^**						
Self	643	626	10.11 (9.25–10.97)	9.49 (8.59–10.39)	13.12 (9.67–16.56)	12.64 (9.23–16.05)
Other household member	480	504	8.12 (7.29–8.96)	8.15 (7.23–9.08)	6.85 (4.27–9.44)	9.41 (6.28–12.44)
No	5,089	5,067	81.77 (80.63–82.91)	82.36 (81.13–83.59)	80.03 (75.95–84.12)	77.96 (73.64–82.27)
Frequently enjoy attending sporting events**						
No	4,939	5,058	79.64 (78.45–80.83)	79.93 (78.65–81.22)	74.74 (70.37–79.11)	82.76 (78.69–86.83)
Yes	1,418	1,293	20.36 (19.17–21.55)	20.07 (18.78–21.35)	25.26 (20.89–29.63)	17.24 (13.17–21.31)

**Abbreviations**: CI = confidence interval.

* Panelists were asked: “In the past 12 months, how often did you wear hearing protection devices (ear plugs, ear muffs) when attending a loud athletic or entertainment event?”

^†^ Persons who identified as white, black, Asian, or other or multiracial were all non-Hispanic. Persons who identified as Hispanic might be of any race.

^§^ *Northeast:* Connecticut, Maine, Massachusetts, New Hampshire, New Jersey, New York, Pennsylvania, Rhode Island, and Vermont. *Midwest:* Illinois, Indiana, Iowa, Kansas, Michigan, Minnesota, Missouri, Nebraska, North Dakota, Ohio, South Dakota, and Wisconsin. *South:* Alabama, Arkansas, Delaware, District of Columbia, Florida, Georgia, Kentucky, Louisiana, Maryland, Mississippi, North Carolina, Oklahoma, South Carolina, Tennessee, Texas, Virginia, and West Virginia. *West:* Alaska, Arizona, California, Colorado, Hawaii, Idaho, Montana, Nevada, New Mexico, Oregon, Utah, Washington, and Wyoming.

^¶^ Panelists were asked: “Do you, or does anyone in your household have deafness or hard of hearing in either ear?”

** Panelists were asked: “Which of the following leisure-time activities do you frequently enjoy doing?” Responses included “Attending sporting events.”

Compared with adults who had a bachelor’s degree or other higher education, those with a high school education or less (odds ratio [OR] = 1.7) and those with some college education (OR = 1.6) were significantly more likely to not wear HPDs (Table 2). Adults aged ≥35 years were significantly more likely to not wear HPDs than were young adults aged 18–24 years. Among adults who frequently enjoy attending sporting events as a leisure-time activity, women were twice as likely (OR = 2.0) as men to seldom or never wear HPDs. Adults with hearing impairment or with a deaf or hard-of-hearing household member were significantly more likely to wear HPDs than were those without hearing impairment in a household member or themselves.

**TABLE 2:** Adjusted multinomial logistic regression comparing frequencies of use of personal hearing protection devices (HPDs) when attending a loud athletic or entertainment event in the past 12 months among adults aged ≥18 years — Porter Novelli SpringStyles panelists, United States, 2018

Characteristic	Comparison of less frequent and more frequent use of personal HPDs*			
	All respondents	All respondents	Frequently attending sporting event^†^	
	Never/Seldom versus Most/Always	Some/Half versus Most/Always	Never/Seldom versus Most/Always	Some/Half versus Most/Always
	OR (95% CI)	OR (95% CI)	OR (95% CI)	OR (95% CI)
**Sex**				
Men	Referent	Referent	Referent	Referent
Women	1.85^§^ (1.54–2.24)	1.05 (0.83–1.33)	2.04^§^ (1.25–3.31)	1.25 (0.71–2.22)
**Age group (yrs)**				
18–24	Referent	Referent	Referent	Referent
25–34	1.26 (0.90–1.77)	0.99 (0.65–1.49)	0.52 (0.22–1.24)	0.46 (0.17–1.23)
35–44	1.46^§^ (1.03–2.08)	1.17 (0.76–1.79)	0.65 (0.27–1.60)	0.56 (0.20–1.55)
45–54	1.48^§^ (1.05–2.10)	0.75 (0.48–1.17)	1.31 (0.48–3.57)	0.45 (0.14–1.47)
55–64	1.48^§^ (1.04–2.10)	0.83 (0.54–1.28)	0.63 (0.25–1.59)	0.50 (0.18–1.42)
65–74	1.57^§^ (1.09–2.26)	0.80 (0.50–1.28)	0.78 (0.28–2.13)	0.54 (0.17–1.74)
≥75	2.59^§^ (1.53–4.37)	0.71 (0.35–1.47)	1.52 (0.27–8.68)	1.10 (0.15–7.93)
**Race/Ethnicity^¶^**				
White	Referent	Referent	Referent	Referent
Black	1.25 (0.91–1.73)	1.75^§^ (1.19–2.58)	2.24 (0.87–5.77)	1.80 (0.61–5.29)
Hispanic	1.07 (0.81–1.42)	1.69^§^ (1.19–2.39)	0.46^§^ (0.25–0.85)	1.00 (0.48–2.08)
Asian	2.93^§^ (1.66–5.16)	2.22^§^ (1.13–4.37)	5.75 (0.47–71.08)	7.23 (0.53–99.32)
Other, multiracial	1.23 (0.79–1.91)	1.69 (0.99–2.89)	9.27 (0.44–197.17)	28.00 (1.28–613.33)
**Education**				
Bachelor's degree or higher	Referent	Referent	Referent	Referent
High school or less	1.69^§^ (1.32–2.16)	1.41^§^ (1.02–1.95)	0.95 (0.52–1.74)	0.89 (0.42–1.85)
Some college or associate degree	1.61^§^ (1.26–2.06)	1.52^§^ (1.11–2.09)	1.62 (0.86–3.05)	1.93 (0.93–4.01)
**Income**				
<$40,000	Referent	Referent	Referent	Referent
$40,000–$74,999	1.10 (0.85–1.43)	0.74 (0.53–1.03)	1.14 (0.58–2.25)	1.14 (0.49–2.63)
$75,000–$124,999	0.95 (0.73–1.24)	0.75 (0.54–1.05)	2.56^§^ (1.25–5.21)	2.16 (0.94–4.99)
≥$125,000	1.34 (0.98–1.84)	1.26 (0.85–1.86)	1.85 (0.86–3.98)	2.09 (0.85–5.18)
U.S. Census region of residence**				
Northeast	Referent	Referent	Referent	Referent
Midwest	0.94 (0.67–1.30)	1.05 (0.69–1.58)	0.72 (0.32–1.62)	0.69 (0.26–1.78)
South	0.69^§^ (0.52–0.91)	0.79 (0.55 – 1.14)	0.47 (0.22–1.00)	0.78 (0.33–1.86)
West	0.61^§^ (0.45–0.83)	0.76 (0.52–1.13)	0.58 (0.25–1.34)	0.44 (0.16–1.17)
**Metropolitan statistical area status**				
Nonmetropolitan	Referent	Referent	Referent	Referent
Metropolitan	1.38^§^ (1.07–1.78)	1.14 (0.82–1.59)	1.77 (0.92–3.39)	1.22 (0.56–2.67)
**Household hearing impairment^††^**				
No	Referent	Referent	Referent	Referent
Yes	0.66^§^ (0.49–0.90)	1.24 (0.85–1.82)	0.35^§^ (0.17–0.71)	0.56 (0.24–1.32)
Other household member	0.70^§^ (0.50–0. 97)	0.73 (0.47–1.13)	0.52 (0.24–1.11)	0.53 (0.21–1.35)
**Frequently enjoy attending sporting events^†^**				
No	Referent	Referent	**—**	**—**
Yes	1.40^§^ (1.09–1.79)	1.68^§^ (1.24–2.27)	**—**	**—**

**Abbreviations**: CI = confidence interval; OR = odds ratio.

* Panelists were asked: “In the past 12 months, how often did you wear hearing protection devices (ear plugs, ear muffs) when attending a loud athletic or entertainment event?”

^†^ Panelists were asked: “Which of the following leisure-time activities do you frequently enjoy doing?” Responses included “Attending sporting events.”

^§^ Statistical difference at p<0.05 compared with the referent group.

**^¶^** Persons who identified as white, black, Asian, or other or multiracial were all non-Hispanic. Persons who identified as Hispanic might be of any race.

** *Northeast:* Connecticut, Maine, Massachusetts, New Hampshire, New Jersey, New York, Pennsylvania, Rhode Island, and Vermont. *Midwest:* Illinois, Indiana, Iowa, Kansas, Michigan, Minnesota, Missouri, Nebraska, North Dakota, Ohio, South Dakota, and Wisconsin. *South:* Alabama, Arkansas, Delaware, District of Columbia, Florida, Georgia, Kentucky, Louisiana, Maryland, Mississippi, North Carolina, Oklahoma, South Carolina, Tennessee, Texas, Virginia, and West Virginia. *West:* Alaska, Arizona, California, Colorado, Hawaii, Idaho, Montana, Nevada, New Mexico, Oregon, Utah, Washington, and Wyoming.

^††^ Panelists were asked: “Do you, or does anyone in your household have deafness or hard of hearing in either ear?”

## Discussion

In this analysis, approximately 8% of participants reported consistent use of an HPD at loud athletic or entertainment events. Approximately two thirds of adults who were more likely to wear an HPD had at least some college education, and approximately half had higher income levels. Women and older adults were significantly less likely to wear an HPD.

Persons with auditory damage caused by excessive loud sound exposure often do not recognize it. An analysis of 2011–2012 data from the National Health and Nutrition Examination Survey found that one in four U.S. adults who reported excellent or good hearing had damage to their hearing suggestive of excessive exposure to loud sounds (*2*). During a given 24-hour period, persons are exposed to a wide range of loud sounds, including not only those at work, but also at home, school, and places of recreation, thereby complicating the determination of an exposure level that would provide an adequate level of safety to protect hearing.

It has been reported that despite an apparent understanding of the effects of noise exposure from loud activities, much of the public appears unconcerned about the use of HPDs during recreational activities (*9*). As part of a health belief model, a construct to describe factors that affect participation in a health behavior and personal experience of noise injury symptoms, as well as awareness of the benefits of ear plugs and the long-term implications of hearing damage are key motivators for using HPDs (*10*).

The findings in this report are subject to at least two limitations. First, the data obtained in this survey were self-reported and relied on respondents’ perceptions of loudness, recall of attendance at events, and their HPD use. Second, although a subgroup of panelists reported frequently enjoying sporting events, that frequency was not defined, and frequency of attending was interpreted by the respondent.

The reported infrequent use of HPDs at loud athletic and entertainment events suggests the need for an increased public health focus on recreational noise exposure, including efforts to raise awareness about the adverse health effects of excessive noise exposure at home and in recreational settings, as well as the protective value of HPDs. Discussions between patients and health care providers regarding the consequences of excessive sound exposure and the potential benefits to health from the use of hearing protection might provide opportunities to prevent or reduce harmful effects.^¶^

**Summary**

**What is already known about this topic?**

Noise-induced hearing loss is a substantial, often unrecognized, health problem.

**What is added by this report?**

Among surveyed U.S. adults, approximately 8% reported consistent use of a hearing protection device (HPD) at loud athletic or entertainment events; women and older adults were less likely to use an HPD, whereas adults with hearing impairment, or who had a hearing-impaired household member, or some college education were significantly more likely to use an HPD.

**What are the implications for public health practice?**

Increasing awareness about the adverse health effects of excessive noise exposure and the simple preventive measures to reduce risk are needed. Health care providers can help their patients prevent or reduce the risks for noise-induced hearing loss.

## Footnotes

* https://nepis.epa.gov/Exe/ZyPDF.cgi/2000L3LN.PDF?Dockey=2000L3LN.PDF.

† http://www.who.int/iris/handle/10665/66217.

§
https://www.gfk.com/fileadmin/user_upload/dyna_content/US/documents/KnowledgePanel_-_A_Methodological_Overview.pdf .

¶
https://www.nap.edu/catalog/23446/hearing-health-care-for-adults-priorities-for-improving-access-and .
